# The Effect of Low-level Laser Therapy on Trigeminal Neuralgia: A Review of Literature

**DOI:** 10.5681/joddd.2014.001

**Published:** 2014-03-05

**Authors:** Farnaz Falaki, Amir Hossein Nejat, Zohreh Dalirsani

**Affiliations:** ^1^Assistant Professor of Oral Medicine, Oral & Maxillofacial Diseases Research Center, Faculty of Dentistry, Mashhad University of Medical Sciences, Mashhad, Iran; ^2^Dentist, Faculty of Dentistry, Mashhad University of Medical Sciences, Mashhad, Iran; ^3^Associate Professor of Oral Medicine, Dental Research Center, Faculty of Dentistry, Mashhad University of Medical Sciences, Mashhad, Iran

**Keywords:** Trigeminal nerve, neuralgia, laser, low level, review

## Abstract

The effect of low intensity laser radiation in the treatment of acute and chronic pain is now established in many studies. Tri-geminal neuralgia is a pain passes through nerve's branches and its trigger is located in skin or mucosa that could lead to pain with a trigger stimulus. The pain involved branches of trigeminal nerve that sometimes has patients to seek the treatment for several years. Nowadays different treatments are used for relief of pain that most of them cause tolerance and various side effects. This paper reviews and summarizes scientific papers available in English literature publishedin PubMed, Scopus, Science Direct, Inter science, and Iran Medex from 1986 until July 2011 about the effect of these types of lasers on trigeminal neuralgia which is one of the most painful afflictions known. In different studies, the effect of laser therapy has been compared with placebo irradiation or medicinal and surgical treatment modalities. Low-level laser therapy (LLLT) is a treatment strategy which uses a single wavelength light source. Laser radiation and monochromatic light may alter cell and tissue function. However, in most studies laser therapy was associated with significant reduction in the intensity and frequency of pain compared with other treatment strategies, a few studies revealed that between laser and placebo group there was not any significant difference according to the analgesic effect. Low-level laser therapy could be considered in treatment of trigeminal neuralgia without any side effects.

## Introduction

Although trigeminal neuralgia *(Tic douloureux)* is one of the most painful conditions which are often reported in patients older than 50 years of age, it may affect younger people or even children. It is estimated that 1 in 15,000 people suffers from trigeminal neuralgia; however, numbers may be significantly higher due to frequent misdiagnosis.^1^

Trigeminal neuralgia (TN) is mainly a periodic, unilateral, sharp, and electric shock-like pain which passes through trigeminal nerve branches and feels in the eyes, lips, nose, scalp, forehead, and jaw and is limited to one side of the face in majority of cases (95%).^1^

The episodes of pain last up to two minutes and between two episodes the patient is painless. It may occur spontaneously or be triggered by daily activities, such as washing, shaving, talking, brushing teeth, eating and drinking. This pain usually takes only a few seconds. Its frequency varies from a single attack during the day to more than one attack per minute that affects the individual’s quality of life.^1^

The most commonly involved branches are maxillary or mandibular division and the least frequently involved is ophthalmic branch.^2^ Pain usually starts after stimulation of trigger points. Some normal activities such as chewing and speaking are common triggers for beginning of the pain.^2^

Different methods have been used for relief of the pain, and the aim of this study was to review studies on the use of laser therapy for trigeminal neuralgia as a new strategy with few side effects. Etiology and pathogenesis as well as management of trigeminal neuralgia are also described.

###  Etiology and Pathogenesis

The exact etiology and pathogenesis of TN is not clearly understood. However, theories of the TN pathogenicity are divided to peripheral and central. Peripheral theories are based on peripheral axon and myelin changes which lead to chemical and physical stimuli threshold changes. Central pathogenecity theories are based on similarity between TN and focal epilepsy due to pressure on nerve branches or ganglia.^2,3^

Some researchers believe both central and peripheral mechanisms are present. For example, pressure on entering branches of brain stem may lead to myelin injury, nerve demylination, abnormal depolarization, ectopic pulses, and pain.^1^

There are certain factors that can trigger the onset of TN such as compression of the nerve (intracranial and extra cranial tumors or vascular anomaly), demyelinating conditions (such as multiple sclerosis—MS), infectious agents (human herpes simplex virus), and nerve trauma.^1^ Traumatic accidents, dental treatment, and various infections could cause trigeminal nerve injury. Some studies revealed that an important reason for primary onset is artery pressure on the nerve, as superior cerebellar artery may irritate the trigeminal nerve.^3,4^

###  Management

When the cause of the TN is identifiable, treatment involves elimination of the cause. In idiopathic cases, however, a variety of medicinal and surgical treatment modalities should be considered. Medicinal management includes carbamazepine, phenytoin, baclofen, gabapentin, oxcarbazepine, lamotrigine, pimozide, and tizanidine hydrochloride.^1,5^

Although medication is the first line of treatment, tolerance may develop as the treatment period and the need for extra dosage increase, which leads to more side effects. Nearly 50% of TN sufferers are not satisfied with medical therapy, because of incomplete control of pain or drug-related side effects. Drowsiness, fatigue, dizziness, nausea, nystagmus, memory loss, and a sense of exhaustion are common side effects of carbamazepine which necessitates finding alternative treatment.^1,6^

Other management which is indicated when medication is not useful include peripheral nerve branch procedures (e.g. peripheral alcohol injection, peripheral neurectomy), percutaneous procedures, percutaneous radiofrequency thermocoagulation (coagulation), glycerol injections, and balloon compression.^6^

Open surgical procedures include trigeminal root section, sectioning of the trigeminal tract in the lower medulla, and microvascular decompression surgery.^6^ Newly proposed treatment methods include microneurovascular decompression, radiofrequency coagulation, gamma knife stereotactic radiosurgery, cryosurgery, and laser therapy.^6-16^

Surgical treatments are considered when drug therapy fails in controlling of TN pain. The results depend on the experience, expertise, and correct selection of technique of neurosurgery team.

### Low-level Laser Therapy (LLLT) 

Low-level laser therapy (LLLT) is a treatment strategy which uses a single wavelength light source. Laser radiation and monochromatic light may alter cell and tissue function.^17,18^ Many authors have reported significant pain reduction in a number of conditions such as rheumatoid arthritis, fibromyalgia, post-operative pain, headache, nervous system diseases, myofascial pain syndrome, chronic neck pain, and low back pain as a result of laser application.^17,19-23^

Clinical studies of the effects of LLLT on injured nerves have revealed an increase in nerve function and improved capacity for myelin production. LLLT has also been shown to be effective for promoting axonal growth in injured nerves in animal models.^18,21,23,24^

Here we review papers available about the effect of low-level lasers on trigeminal neuralgia. An online search of PubMed, Scopus, Science Direct, Inter science, and Iran Medex using key words “trigeminal neuralgia” and “low-level laser” from 1986 until July 2011 was performed. Studies with methods not including the exact details of laser therapy were excluded from the review.

### Review of Articles 

In one study, 18 outpatients suffering severe post-herpetic neuralgia (PHN) were exposed to low-power helium–neon (He-Ne) laser.^25^ The investigators used four-grade estimation, visual analog scale (VAS), and modified McGill pain questionnaire (m-MPQ) after every 10 of 50 irradiations to determine efficacy of treatment. Results showed that repeated irradiation with low-power He-Ne laser is an effective and safe therapy for post-herpetic neuralgia (PHN).^25^

In another study by Iijima et al,^23^ 36 patients suffering from PHN were exposed to low-power He-Ne laser 2 or 3 times a week. The results showed that the efficacy of the laser at the end of 20 trials was 88.9%, and the degree of pain relief was 55.3%.

Walker^26^ used low power He-Ne laser (1 mW, 632.5 nm, 20 Hz) in patients with chronic pain. He treated 26 patients with trigeminal neuralgia, post-herpetic neuralgia, sciatica, and osteoarthritis. He irradiated the affected sites 30 to 90 seconds three days per week for 10 consecutive weeks. Nineteen patients revealed significant reduction in frequency and also intensity of their pain after the treatment duration.^26^

In another study, Walker et al. exposed the patients with the history of TN with He-Ne laser (1 mW, 632.5 nm, 20 Hz) in a double blind study. Duration of exposure for each site was 20 seconds for skin overlying peripheral nerves and painful facial areas. The control group received no irradiation with a laser apparatus identical to the laser group. Both groups received irradiation three times a week for ten weeks. Laser therapy was associated with significant reduction in the intensity and frequency of pain.^24^

Eckedral designed a double-blind, placebo controlled study to investigate effectiveness of low-level laser therapy (LLLT) on TN in Denmark. Sixteen patients suffering from TN were radiated with laser for 5 weeks (830 nm, 30 mW) and compared with 14 patients as control group. After one year follow-up they got to the point that LLLT is an effective method and an excellent supplement to conventional methods for TN therapy.^27^

Moore et al.^28^ performed a double-blind cross-over trial of low-level laser therapy (LLLT) in treatment of established PHN of at least 6 months duration with no response to conventional methods of treatment. Patients were treated with a gallium aluminium arsenide (GaAlAs) diode-laser (830 nm: 60mW). Results demonstrated a significant reduction in the intensity and distribution of pain (74% of patients) due to a course of LLLT (4 consecutive laser treatments).

Samosiuk et al.^29^ divided 137 patients with typical TN in 4 groups. Thirty patients (G1) received EHF therapy (extremely high frequency puncture), 30 patients exposed to laser (G2), 67 patients treated with combination of laser and EHF-puncture (G3), 10 patients were in control group received physiotherapy. All patients were given carbamazepine. Best results were given from G3 which 31% of cases could stop carbamazepine and the rest of them could reduce its dose by 50-70%.

Vernon and Hasbun reported a 61-year-old female with TN. They treated her with GaAlAs diode-laser (808 nm, 200mW) with protocol of every day irradiation with two days interval after each five day. Total of 20 sessions of exposure performed. The patient reported no pain at the 12^th^ session. ^30^

Kemmotsu et al^31^ evaluated the efficacy of low -level laser in 63 patients with PHN. They used GaAlAs laser (830 nm, 60 mW continuous wave) as LLLT system. The immediate effect after the initial LLLT was very good and good in 26 and 30 patients, respectively. The long term effect after completion of exposure period resulted in no pain in 12 cases and slight pain in 46 patients. In this study, no complications and side effects was reported.

Kim et al^32^ used He-Ne, GaAlAs and CO_2_ laser in patients suffering from trigeminal neuralgia. Twenty five patients divided in two groups: one group treated with low-level laser therapy and another group treated with laser and medication. Visual analogue scale showed that compared with the combined therapy, the laser therapy alone cause more pain relief.

Mann et al.^33^33 investigated the efficacy of combi-laser in 50 patients with PHN. In each patient the laser therapy was given for 15 days with the period of 5 minutes and 6 seconds exposure for each appointment. The therapeutic effect of the laser was evaluated after 5^th^ and 10^th^ and 15^th^ day with visual analogue scale (VAS). Patients started responding after third day and after completion of therapy, 43 out of 50 showed great relief (76-100%), four cases showed good relief (51-75%), two cases with fair relief (26-50%) and one case with poor relief (1-25%).^33^

Romaniello et al.^34^ reported that because of high density of receptors in the facial skin and short distances of conductions in comparison with limb extremities, laser evoked potential (LEP) recordings after trigeminal stimulation are easier and quicker. They represented that oral mucosa, upper lip, skin of supra-orbital region, chin and temples are sensitive areas to laser irradiation.

In contrast to most studies, another study revealed that between laser and placebo irradiation groups there was not any statistically significant difference according to the analgesic effect.^35^

## Discussion

However few studies have been performed on effect of low -level laser therapy on trigeminal neuralgia, regarding the results of the most studies and the pain relief effect of laser on chronic pains, it seems that laser therapy could be an appropriate substitute for current treatments in which side effects are really annoying for the patient.

Among Studies, two studies by Ijima et al^23,25^ used He-Ne laser which resulted in favorable reduction in PHN after both 20 and 50 sessions. Walker found that He-Ne laser was effective in patients with trigeminal neuralgia, post-herpetic neuralgia, sciatica, and osteoarthritis after 30 sessions of irradiation.^26^ In addition, in another study He-Ne laser was used in treatment of TN with wavelength of 632.5 nm and power of 1 mW for 30 sessions and found it useful after one-year follow up.^24^

In the mentioned studies, each point had received 6 to 10 J in chronic forms and 3 to 6 J in acute ones. Laser had been radiated in various points: trigger points, nerve pass out of bone, and also acupuncture points. No study has been performed to compare different points of radiation. Simunovic believes that He-Ne laser is the most proper laser for TN and radiation in trigger points is more effective than any other points.^36^

Effectiveness of LLLT in resistant PHN was also found. In that study, they used GaAlAs with wavelength of 830 nm and power of 60mW. They reached favorable results after 4 sessions which is less than treatment with He-Ne laser.^28^ Kemmotsu et al^31^ also used GaAlAs and irradiated laser for 36 sessions on average and demonstrated good long-term results in resistant PHN cases. Vernon and Hasbun^30^ used GaAlAs laser to treat a patient with TN. They found LLLT very effective in pain relief after 12 sessions. However, they completed 20 sessions of irradiation. Mann et al^33^ also reported great pain relief after 15 radiation sessions with combi-laser (1000Hz and 12x70 watts). In addition, Mittal et al^37^ used combi-laser (5000 Hz and 8 J/cm^2^) for16 sessions and demonstrated the same results.

Different mechanisms have been considered for the pain attenuation of the low-level lasers include effect on prostaglandin (PG) synthesis, increase in the change of PG type G and PG type H_2_ into PG type I_2_, increase in beta-endorphins level in CSF, increase in glucocorticoids urinary secretion (glucocorticoids are beta-endorphin synthesis inhibitor), increase in pain threshold in nerve fibers, increase in serotonin urinary secretion, decrease in histamine and serotonin secretion, decrease in bradykinin synthesis (pain inducer substance), change in norepinephrine and epinephrine activity, increase in ATP production, increase in local microcirculation, lymph node circulation enhancement and edema decline.^38-40^

Also, LLLT can modulate inflammatory pain by reducing levels of biochemical markers (PGE2, mRNA Cox 2, IL-1β, TNF-α), neutrophil cell influx, oxidative stress, and formation of edema and hemorrhage in a dose-dependent manner.^39,41^

It should be mentioned that distinguishing neuralgia from other chronic pains such as atypical facial pain plays an important role in effectiveness of treatment.

Briefly, however, studies about low-level laser therapy on trigeminal neuralgia are various according to numbers of sessions and wavelengths and duration of irradiations, most of them demonstrated effectiveness of laser therapy in pain relief. A study is recommended for comparison of different methods for finding the most effective properties of laser therapy on trigeminal neuralgia.

## Conclusion

The studies revealed that low-level laser therapy could be considered in treatment of trigeminal neuralgia. Laser causes pain relief without any side effects. It could be helpful especially in patients suffering from neuralgia tolerated to drug therapy. It should be mentioned that distinguishing neuralgia from other chronic pain such as atypical facial pains is important in effectiveness of treatment.
